# The genome sequence of the centipede *Strigamia acuminata *(Leach, 1816)

**DOI:** 10.12688/wellcomeopenres.19941.1

**Published:** 2023-09-21

**Authors:** Gregory D. Edgecombe, Duncan Sivell, Natural History Museum Genome Acquisition Lab, Darwin Tree of Life Barcoding collective, Wellcome Sanger Institute Tree of Life programme, Wellcome Sanger Institute Scientific Operations: DNA Pipelines collective, Tree of Life Core Informatics collective, Darwin Tree of Life Consortium

**Affiliations:** 1 Natural History Museum, London, England, UK

**Keywords:** Strigamia acuminata, centipede, genome sequence, chromosomal, Geophilomorpha

## Abstract

We present a genome assembly from an individual male *Strigamia acuminata* (centipede; Arthropoda; Chilopoda; Geophilomorpha; Geophilidae; Linotaeniinae). The genome sequence is 237.5 megabases in span. Most of the assembly is scaffolded into 11 chromosomal pseudomolecules, including the X and Y sex chromosomes. The mitochondrial genome has also been assembled and is 15.07 kilobases in length.

## Species taxonomy

Eukaryota; Metazoa; Eumetazoa; Bilateria; Protostomia; Ecdysozoa; Panarthropoda; Arthropoda; Mandibulata; Myriapoda; Chilopoda; Pleurostigmophora; Epimorpha; Geophilomorpha; Geophilidae; Linotaeniinae; *Strigamia*; *Strigamia acuminata* ( Leach, 1816) (NCBI:txid1255758).

## Background

*Strigamia acuminata* is a western Palaearctic species distributed throughout most of continental Europe, from the Iberian Peninsula to the Caucasus and Volga basin, as well as Sicily, Crete and Great Britain ( Bonato *et al.*, 2012; Bonato *et al.*, 2016; Bonato *et al.*, 2023). Integrative taxonomy suggests that European populations of *S. acuminata* form a complex of at least two species ( Bonato *et al.*, 2023). In the UK, *S. acuminata* is known only from England and Wales, the largest numbers of records being from the southeast and east of England. The following occurrence data are summarised from Barber (2022). It is usually an inland species although some coastal records are known. It is collected throughout the year but has a patchy and unpredictable occurrence. Records are overwhelmingly in rural sites, with only a few urban occurrences. In Britain, as in Europe, *S. acuminata* is most common in deciduous woodland, and is less frequently found in mixed woodland and wetland. Its most typical microsites are under dead wood or in leaf litter. Most collections are from lowland sites (<200 m), but it has been found at up to 1,013 m in Snowdonia, north Wales.

Like the other two species of *Strigamia* in the UK, *S. acuminata* is distinguished in the field from most other British Geophilomorpha by its reddish colouration. Adults reach a body length of 30 mm in England and Wales but up to 40 mm in some European populations. A leg number of 37 to 41 pairs (versus 47 or more pairs) provides the simplest basis for its distinction from the other two British congeners, *S. crassipes* and *S. maritima* ( Barber, 2008). Males have 37 or 39 leg pairs and females 41 in the UK; in other parts of the geographic range 39–43 pairs encompass the variation ( Bonato *et al.*, 2023). As in other congeners, the ultimate legs of females are slender and those of males swollen. In Germany, *S. acuminata* has been observed to group feed on the millipede *Julus* ( Weil, 1958). 

Few whole genome sequences for centipedes have been generated, including those of *Strigamia maritima* (GCA_000239455.1) ( Chipman *et al.*, 2014) and of *Lithobius niger* (GCA_023313725.1), *Rhysida immarginata* (GCA_023313115.1) and *Thereuonema tuberculata* (GCA_023159025.1) ( So *et al.*, 2022). The MetaInvert database at Senckenberg Görlitz and the LOEWE Centre for Translational Biodiversity Genomics offers draft genome sequences for a broad spectrum of soil invertebrates, encompassing 19 Chilopoda, including *S. acuminata*, aiming to extend the taxonomic scope for soil metagenomic studies ( Collins *et al.*, 2023).

The genome of *Strigamia acuminata* was sequenced as part of the Darwin Tree of Life Project, a collaborative effort to sequence all named eukaryotic species in the Atlantic Archipelago of Britain and Ireland. Here we present a chromosomally complete genome sequence for *Strigamia acuminata*, based on one adult male specimen from Bookham Common, Surrey. The genome is of interest for comparison with the most comprehensively annotated centipede genome, and the first to be sequenced, the closely related *S. maritima* ( Chipman *et al.*, 2014), which inhabits the littoral zone rather than woodland.

## Genome sequence report

**Figure 1.**
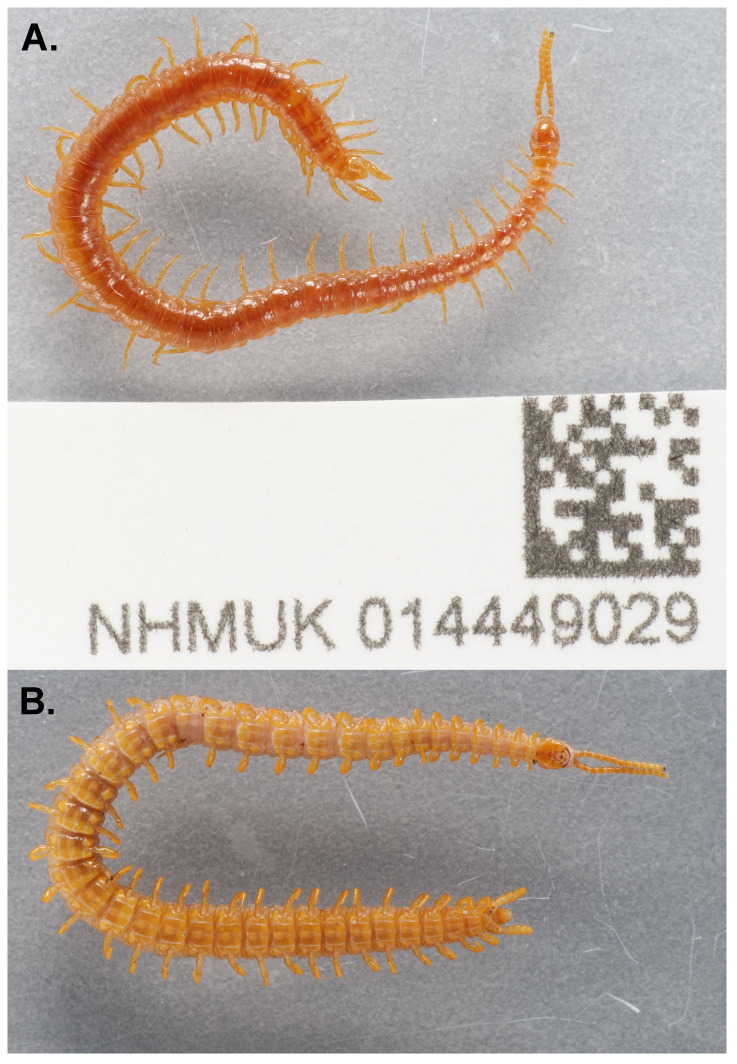
Photograph of the *Strigamia acuminata* specimen (NHMUK014449029) used for genome sequencing. **A**, dorsal view; **B**, ventral view.

The genome was sequenced from one adult male *Strigamia acuminata* ( Figure 1) collected from Bookham Common, Leatherhead, England (51.29, –0.38). A total of 98-fold coverage in Pacific Biosciences single-molecule HiFi long reads was generated. Primary assembly contigs were scaffolded with chromosome conformation Hi-C data. Manual assembly curation corrected 99 missing joins or mis-joins and removed 23 haplotypic duplications, reducing the assembly length by 1.68% and the scaffold number by 27.06%, and increasing the scaffold N50 by 48.44%.

**Figure 2.**
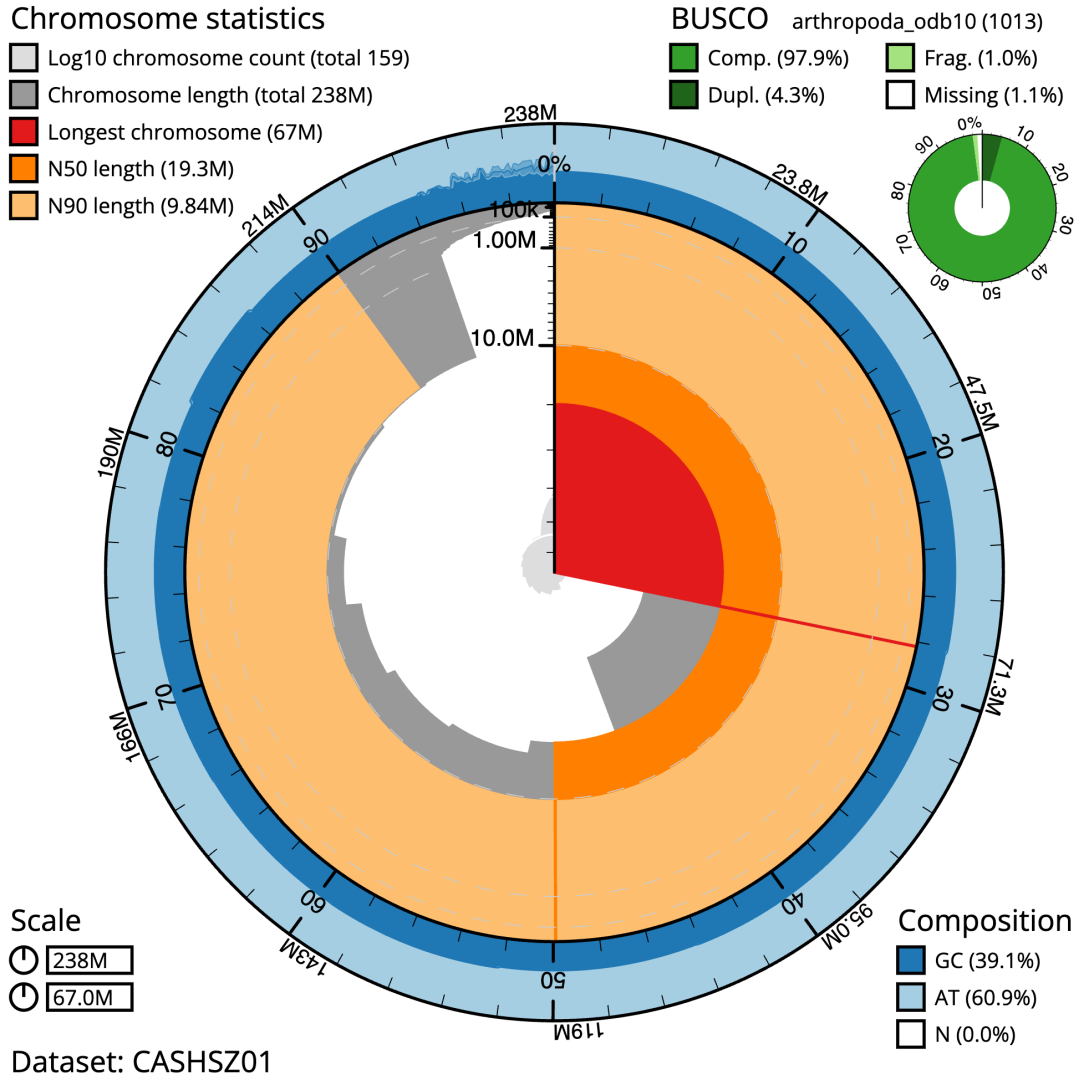
Genome assembly of *Strigamia acuminata*, qcStrAcum1.1: metrics. The BlobToolKit Snailplot shows N50 metrics and BUSCO gene completeness. The main plot is divided into 1,000 size-ordered bins around the circumference with each bin representing 0.1% of the 237,534,282 bp assembly. The distribution of scaffold lengths is shown in dark grey with the plot radius scaled to the longest scaffold present in the assembly (66,985,994 bp, shown in red). Orange and pale-orange arcs show the N50 and N90 scaffold lengths (19,347,077 and 9,836,499 bp), respectively. The pale grey spiral shows the cumulative scaffold count on a log scale with white scale lines showing successive orders of magnitude. The blue and pale-blue area around the outside of the plot shows the distribution of GC, AT and N percentages in the same bins as the inner plot. A summary of complete, fragmented, duplicated and missing BUSCO genes in the arthropoda_odb10 set is shown in the top right. An interactive version of this figure is available at https://blobtoolkit.genomehubs.org/view/qcStrAcum1.1/dataset/CASHSZ01/snail.

The final assembly has a total length of 237.5 Mb in 158 sequence scaffolds with a scaffold N50 of 19.3 Mb ( Table 1). Most (94.83%) of the assembly sequence was assigned to 11 chromosomal-level scaffolds, representing 9 autosomes and the X and Y sex chromosomes. Chromosome-scale scaffolds confirmed by the Hi-C data are named in order of size ( Figure 2– Figure 5; Table 2). X and Y chromosomes in this genus are reported to be homomorphic ( Green *et al.*, 2016). In this assembly, SUPER_X and SUPER_Y both have half coverage. Y was determined based on the absence of BUSCO genes (arthropoda-odb10 gene set). While not fully phased, the assembly deposited is of one haplotype. Contigs corresponding to the second haplotype have also been deposited. The mitochondrial genome was also assembled and can be found as a contig within the multifasta file of the genome submission.

**Table 1.** Genome data for *Strigamia acuminata*, qcStrAcum1.1.

Project accession data		
Assembly identifier	qcStrAcum1.1	
Species	*Strigamia acuminata*	
Specimen	qcStrAcum1	
NCBI taxonomy ID	1255758	
BioProject	PRJEB59944	
BioSample ID	SAMEA10800140	
Isolate information	qcStrAcum1, male (DNA sequencing and Hi-C data)	
Assembly metrics *		*Benchmark*
Consensus quality (QV)	59.6	*≥ 50*
*k*-mer completeness	99.99%	*≥ 95%*
BUSCO **	C:97.9%[S:93.6%,D:4.3%],F:1.0%, M:1.1%,n:1,013	*C ≥ 95%*
Percentage of assembly mapped to chromosomes	94.83%	*≥ 95%*
Sex chromosomes	X and Y chromosomes	*localised homologous pairs*
Organelles	Mitochondrial genome assembled	*complete single alleles*
Raw data accessions		
PacificBiosciences SEQUEL II	ERR10906091	
Hi-C Illumina	ERR10908620	
Genome assembly		
Assembly accession	GCA_949358305.1	
*Accession of alternate haplotype*	GCA_949357715.1	
Span (Mb)	237.5	
Number of contigs	343	
Contig N50 length (Mb)	1.8	
Number of scaffolds	158	
Scaffold N50 length (Mb)	19.3	
Longest scaffold (Mb)	67.0	

* Assembly metric benchmarks are adapted from column VGP-2020 of “Table 1: Proposed standards and metrics for defining genome assembly quality” from ( Rhie *et al.*, 2021). ** BUSCO scores based on the arthropoda_odb10 BUSCO set using v5.3.2. C = complete [S = single copy, D = duplicated], F = fragmented, M = missing, n = number of orthologues in comparison. A full set of BUSCO scores is available at https://blobtoolkit.genomehubs.org/view/qcStrAcum1.1/dataset/CASHSZ01/busco.

**Figure 3.**
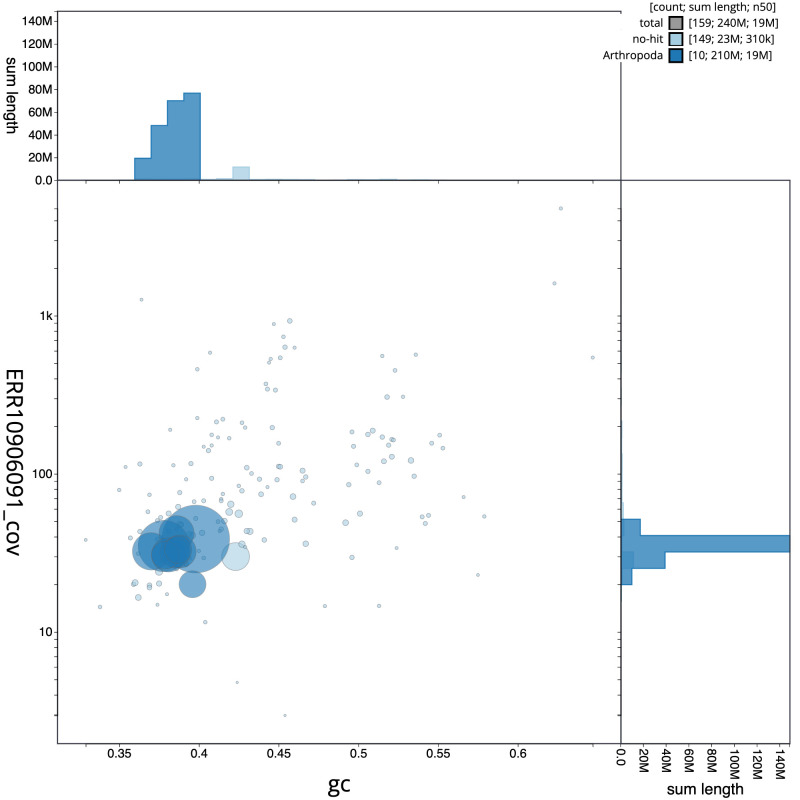
Genome assembly of *Strigamia acuminata*, qcStrAcum1.1: BlobToolKit GC-coverage plot. Scaffolds are coloured by phylum. Circles are sized in proportion to scaffold length. Histograms show the distribution of scaffold length sum along each axis. An interactive version of this figure is available at https://blobtoolkit.genomehubs.org/view/qcStrAcum1.1/dataset/CASHSZ01/blob.

**Figure 4.**
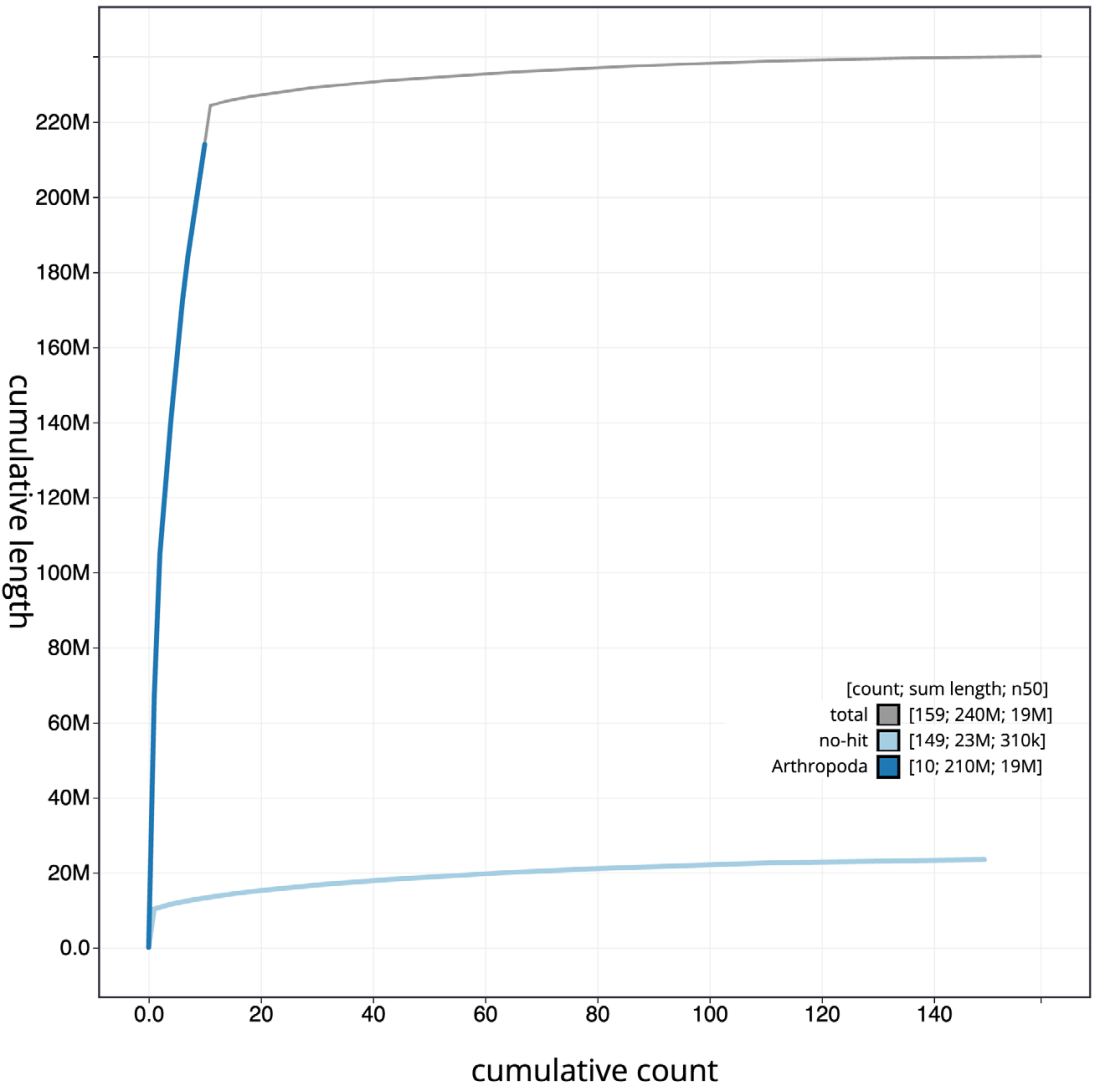
Genome assembly of *Strigamia acuminata*, qcStrAcum1.1: BlobToolKit cumulative sequence plot. The grey line shows cumulative length for all scaffolds. Coloured lines show cumulative lengths of scaffolds assigned to each phylum using the buscogenes taxrule. An interactive version of this figure is available at https://blobtoolkit.genomehubs.org/view/qcStrAcum1.1/dataset/CASHSZ01/cumulative.

**Figure 5.**
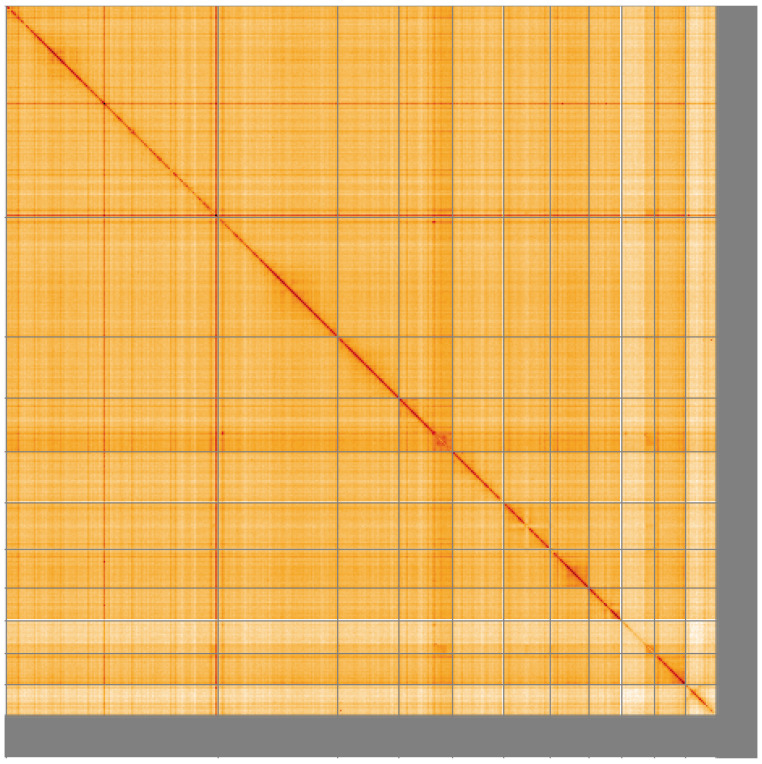
Genome assembly of *Strigamia acuminata*, qcStrAcum1.1: Hi-C contact map of the qcStrAcum1.1 assembly, visualised using HiGlass. Chromosomes are shown in order of size from left to right and top to bottom. An interactive version of this figure may be viewed at https://genome-note-higlass.tol.sanger.ac.uk/l/?d=ePZD50jWQ1uKpCQEifKlEA.

**Table 2.** Chromosomal pseudomolecules in the genome assembly of *Strigamia acuminata*, qcStrAcum1.

INSDC accession	Chromosome	Length (Mb)	GC%
OX442314.1	1	66.99	40.0
OX442315.1	2	37.79	38.0
OX442316.1	3	19.35	37.0
OX442317.1	4	16.99	38.5
OX442318.1	5	16.15	38.0
OX442319.1	6	14.72	39.0
OX442320.1	7	12.25	38.5
OX442321.1	8	10.36	38.0
OX442323.1	9	9.84	38.5
OX442324.1	X	9.63	39.5
OX442322.1	Y	10.34	42.5
OX442325.1	MT	0.02	37.0

The estimated Quality Value (QV) of the final assembly is 59.6 with *k*-mer completeness of 99.99%, and the assembly has a BUSCO v5.3.2 completeness of 97.9% (single = 93.6%, duplicated = 4.3%), using the arthropoda_odb10 reference set ( *n* = 1,013).

Metadata for specimens, spectral estimates, sequencing runs, contaminants and pre-curation assembly statistics can be found at https://links.tol.sanger.ac.uk/species/1255758.

## Methods

### Sample acquisition and nucleic acid extraction

An adult male *Strigamia acuminata* (specimen ID NHMUK014449029, ToLID qcStrAcum1) was collected from Bookham Common, Leatherhead, England, UK (latitude 51.29, longitude –0.38) on 2021-04-20. The specimen was collected by Gregory Edgecombe and Duncan Sivell (both Natural History Museum) and identified by Gregory Edgecombe, and was then preserved in 70% ethanol.

DNA was extracted at the Tree of Life laboratory, Wellcome Sanger Institute (WSI). The qcStrAcum1 sample was weighed and dissected on dry ice with tissue set aside for Hi-C sequencing. Somatic tissue was disrupted using a Nippi Powermasher fitted with a BioMasher pestle. High molecular weight (HMW) DNA was extracted using the Qiagen MagAttract HMW DNA extraction kit. HMW DNA was sheared into an average fragment size of 12–20 kb in a Megaruptor 3 system with speed setting 30. Sheared DNA was purified by solid-phase reversible immobilisation using AMPure PB beads with a 1.8X ratio of beads to sample to remove the shorter fragments and concentrate the DNA sample. The concentration of the sheared and purified DNA was assessed using a Nanodrop spectrophotometer and Qubit Fluorometer and Qubit dsDNA High Sensitivity Assay kit. Fragment size distribution was evaluated by running the sample on the FemtoPulse system.

### Sequencing

Pacific Biosciences HiFi circular consensus DNA sequencing libraries were constructed according to the manufacturers’ instructions. DNA sequencing was performed by the Scientific Operations core at the WSI on a Pacific Biosciences SEQUEL II (HiFi) instrument. Hi-C data were also generated from tissue of qcStrAcum1 using the Arima2 kit and sequenced on the Illumina NovaSeq 6000 instrument.

### Genome assembly, curation and evaluation

Assembly was carried out with Hifiasm ( Cheng *et al.*, 2021) and haplotypic duplication was identified and removed with purge_dups ( Guan *et al.*, 2020). The assembly was then scaffolded with Hi-C data ( Rao *et al.*, 2014) using YaHS ( Zhou *et al.*, 2023). The assembly was checked for contamination and corrected as described previously ( Howe *et al.*, 2021). Manual curation was performed using HiGlass ( Kerpedjiev *et al.*, 2018) and Pretext ( Harry, 2022). The mitochondrial genome was assembled using MitoHiFi ( Uliano-Silva *et al.*, 2023), which runs MitoFinder ( Allio *et al.*, 2020) or MITOS ( Bernt *et al.*, 2013) and uses these annotations to select the final mitochondrial contig and to ensure the general quality of the sequence.

A Hi-C map for the final assembly was produced using bwa-mem2 ( Vasimuddin *et al.*, 2019) in the Cooler file format ( Abdennur & Mirny, 2020). To assess the assembly metrics, the *k*-mer completeness and QV consensus quality values were calculated in Merqury ( Rhie *et al.*, 2020). This work was done using Nextflow ( Di Tommaso *et al.*, 2017) DSL2 pipelines “sanger-tol/readmapping” ( Surana *et al.*, 2023a) and “sanger-tol/genomenote” ( Surana *et al.*, 2023b). The genome was analysed within the BlobToolKit environment ( Challis *et al.*, 2020) and BUSCO scores ( Manni *et al.*, 2021; Simão *et al.*, 2015) were calculated.

Table 3 contains a list of relevant software tool versions and sources.

**Table 3.** Software tools: versions and sources.

Software tool	Version	Source
BlobToolKit	4.1.7	https://github.com/blobtoolkit/blobtoolkit
BUSCO	5.3.2	https://gitlab.com/ezlab/busco
Hifiasm	0.16.1-r375	https://github.com/chhylp123/hifiasm
HiGlass	1.11.6	https://github.com/higlass/higlass
Merqury	MerquryFK	https://github.com/thegenemyers/MERQURY.FK
MitoHiFi	2	https://github.com/marcelauliano/MitoHiFi
PretextView	0.2	https://github.com/wtsi-hpag/PretextView
purge_dups	1.2.3	https://github.com/dfguan/purge_dups
sanger-tol/genomenote	v1.0	https://github.com/sanger-tol/genomenote
sanger-tol/readmapping	1.1.0	https://github.com/sanger-tol/readmapping/tree/1.1.0
YaHS	1.2a	https://github.com/c-zhou/yahs

### Wellcome Sanger Institute – Legal and Governance

The materials that have contributed to this genome note have been supplied by a Darwin Tree of Life Partner. The submission of materials by a Darwin Tree of Life Partner is subject to the **‘Darwin Tree of Life Project Sampling Code of Practice’**, which can be found in full on the Darwin Tree of Life website here. By agreeing with and signing up to the Sampling Code of Practice, the Darwin Tree of Life Partner agrees they will meet the legal and ethical requirements and standards set out within this document in respect of all samples acquired for, and supplied to, the Darwin Tree of Life Project. 

Further, the Wellcome Sanger Institute employs a process whereby due diligence is carried out proportionate to the nature of the materials themselves, and the circumstances under which they have been/are to be collected and provided for use. The purpose of this is to address and mitigate any potential legal and/or ethical implications of receipt and use of the materials as part of the research project, and to ensure that in doing so we align with best practice wherever possible. The overarching areas of consideration are:

• Ethical review of provenance and sourcing of the material

• Legality of collection, transfer and use (national and international) 

Each transfer of samples is further undertaken according to a Research Collaboration Agreement or Material Transfer Agreement entered into by the Darwin Tree of Life Partner, Genome Research Limited (operating as the Wellcome Sanger Institute), and in some circumstances other Darwin Tree of Life collaborators.

## Data Availability

European Nucleotide Archive: *Strigamia acuminata*. Accession number PRJEB59944; https://identifiers.org/ena.embl/PRJEB59944. ( Wellcome Sanger Institute, 2023) The genome sequence is released openly for reuse. The *Strigamia acuminata* genome sequencing initiative is part of the Darwin Tree of Life (DToL) project. All raw sequence data and the assembly have been deposited in INSDC databases. The genome will be annotated using available RNA-Seq data and presented through the Ensembl pipeline at the European Bioinformatics Institute. Raw data and assembly accession identifiers are reported in Table 1.

## Author information

Members of the Natural History Museum Genome Acquisition Lab are listed here: https://doi.org/10.5281/zenodo.4790042.

Members of the Darwin Tree of Life Barcoding collective are listed here: https://doi.org/10.5281/zenodo.4893703.

Members of the Wellcome Sanger Institute Tree of Life programme are listed here: https://doi.org/10.5281/zenodo.4783585.

Members of Wellcome Sanger Institute Scientific Operations: DNA Pipelines collective are listed here: https://doi.org/10.5281/zenodo.4790455.

Members of the Tree of Life Core Informatics collective are listed here: https://doi.org/10.5281/zenodo.5013541.

Members of the Darwin Tree of Life Consortium are listed here: https://doi.org/10.5281/zenodo.4783558.
