# Supplementary material for: The genome sequence of the centipede Strigamia acuminata (Leach, 1816)
Source: Wellcome Open Res. 2023 Sep 21;8:420. [Version 1] doi: 10.12688/wellcomeopenres.19941.1 (PMC10556566; doi:10.12688/wellcomeopenres.19941.1)
Supplement: Peer review history

# Reviewer report: Reviewer response for version 1

**DOI:** 10.21956/wellcomeopenres.22082.r67911

**Published:** 2023-10-17

**Authors:** Ken Kraaijeveld

**Affiliations:** 1 Faculty of Earth and Life Sciences, Institute of Ecological Science, Animal Ecology, Vrije University Amsterdam, Amsterdam, The Netherlands

Deep coverage Hifi reads combined with HiC yield a high-quality genome assembly that will be a valuable resource. The work is well-described and nicely illustrated.

 My only request would be to provide more details on the steps taken during the manual assembly curation, as this would be useful to know for future genome assembly projects.

Are sufficient details of methods and materials provided to allow replication by others?

Partly

Is the rationale for creating the dataset(s) clearly described?

Yes

Are the datasets clearly presented in a useable and accessible format?

Yes

Are the protocols appropriate and is the work technically sound?

Yes

Reviewer Expertise:

Genomics, evolution

I confirm that I have read this submission and believe that I have an appropriate level of expertise to confirm that it is of an acceptable scientific standard.

# Reviewer report: Reviewer response for version 1

**DOI:** 10.21956/wellcomeopenres.22082.r67909

**Published:** 2023-10-05

**Authors:** Maurijn van der Zee

**Affiliations:** 1 Institute of Biology, Leiden University, Sylviusweg, Leiden, The Netherlands

A pretty complete genome of the shorter red centipede

 Data are well described, are accessible, and could be downloaded. It is not entirely clear how the 158 scaffolds were assigned to 11 chromosomes, but I guess this was mainly done using the HiC data. It is of course great that these HiC data are available. This high quality genome is a valuable supplement to the Strigamia maritima genome, and a useful contribution to the scientific community.

Are sufficient details of methods and materials provided to allow replication by others?

Yes

Is the rationale for creating the dataset(s) clearly described?

Yes

Are the datasets clearly presented in a useable and accessible format?

Yes

Are the protocols appropriate and is the work technically sound?

Yes

Reviewer Expertise:

Insect evo-devo, comparative genomics, evolve-and-resequence

I confirm that I have read this submission and believe that I have an appropriate level of expertise to confirm that it is of an acceptable scientific standard.

# Reviewer report: Reviewer response for version 1

**DOI:** 10.21956/wellcomeopenres.22082.r67713

**Published:** 2023-10-05

**Authors:** Jerome H.L Hui

**Affiliations:** 1 Chinese University of Hong Kong, Hong Kong, China

This is an excellent genomic resource! Here, Edgecombe and colleagues report the genome sequence of centipede *Strigamia acuminata *(Leach, 1816). This species is widely recorded in England and Wales. To date, genomic resources for myriapods including centipedes are generally scarce when comparing to other arthropods such as insects and crustaceans. Molecular data of this species are also scarce prior to this report, and are mainly confined to COI sequences deposited to the NCBI database. One great achievement that I personally highly appreciate is that this is the first report of the X and Y chromosomal sequences in centipede! This new genome resource will be very useful for further studies, such as understanding their evolutionary relationships with other arthropods.

 In terms of quality, this genome resource is in also excellent judging from the summary statistics, with high BUSCO numbers, high sequence continuity (scaffold N50), and majority of sequences contained on the 11 pseudochromosomes (i.e. 9 autosomes + X + Y) (plus mitochondrion). To sum up, this is an outstanding and valuable contribution.

Are sufficient details of methods and materials provided to allow replication by others?

Yes

Is the rationale for creating the dataset(s) clearly described?

Yes

Are the datasets clearly presented in a useable and accessible format?

Yes

Are the protocols appropriate and is the work technically sound?

Yes

Reviewer Expertise:

Genomics, evolution, invertebrates

I confirm that I have read this submission and believe that I have an appropriate level of expertise to confirm that it is of an acceptable scientific standard.
